# Apyrase treatment of myocardial infarction according to a clinically applicable protocol fails to reduce myocardial injury in a porcine model

**DOI:** 10.1186/1471-2261-10-1

**Published:** 2010-01-04

**Authors:** Jesper van der Pals, Sasha Koul, Michael I Götberg, Göran K Olivecrona, Martin Ugander, Mikael Kanski, Andreas Otto, Matthias Götberg, Håkan Arheden, David Erlinge

**Affiliations:** 1 Department of Cardiology, Lund University Hospital, Lund, Sweden; 2 Department of Clinical Physiology, Lund University Hospital, Lund, Sweden

## Abstract

**Background:**

Ectonucleotidase dependent adenosine generation has been implicated in preconditioning related cardioprotection against ischemia-reperfusion injury, and treatment with a soluble ectonucleotidase has been shown to reduce myocardial infarct size (IS) when applied prior to induction of ischemia. However, ectonucleotidase treatment according to a clinically applicable protocol, with administration only after induction of ischemia, has not previously been evaluated. We therefore investigated if treatment with the ectonucleotidase apyrase, according to a clinically applicable protocol, would reduce IS and microvascular obstruction (MO) in a large animal model.

**Methods:**

A percutaneous coronary intervention balloon was inflated in the left anterior descending artery for 40 min, in 16 anesthetized pigs (40-50 kg). The pigs were randomized to 40 min of 1 ml/min intracoronary infusion of apyrase (10 U/ml, n = 8) or saline (0.9 mg/ml, n = 8), twenty minutes after balloon inflation. Area at risk (AAR) was evaluated by *ex vivo *SPECT. IS and MO were evaluated by *ex vivo *MRI.

**Results:**

No differences were observed between the apyrase group and saline group with respect to IS/AAR (75.7 ± 4.2% vs 69.4 ± 5.0%, p = NS) or MO (10.7 ± 4.8% vs 11.4 ± 4.8%, p = NS), but apyrase prolonged the post-ischemic reactive hyperemia.

**Conclusion:**

Apyrase treatment according to a clinically applicable protocol, with administration of apyrase after induction of ischemia, does not reduce myocardial infarct size or microvascular obstruction.

## Background

Despite great advances in the treatment of ischemic heart disease, acute myocardial infarction remains the most common cause of death in the western world [1]. Modern therapy of acute myocardial infarction is aimed at reperfusion of the ischemic tissue in order to reduce myocardial infarct size (IS) and the extent of complications [2-6]. Although the final infarct size is reduced by early reperfusion, reperfusion per se may lead to immediate and accelerated injury beyond that which is generated by ischemia alone, a phenomenon referred to as "reperfusion injury" [7,8]. Myocardial ischemia may also damage the endothelium leading to impairment of the microvascular blood flow (microvascular obstruction, MO). In the clinical setting, MO is common and an independent predictor of poorer clinical outcome [9]. Consequently, there is a need for an adjunctive cardioprotective therapy for myocardial salvage beyond what reperfusion therapy alone can provide.

A theoretical possibility is to utilize the adenosine receptor system, which seems to represent an innate protection system in the heart. Extracellular adenosine is mainly generated from a two-step dephosphorylation from adenosine triphosphate (ATP) by a family of enzymes collectively known as ectonucleotidases. ATP is degraded to adenosine monophosphate (AMP) by CD39, also called apyrase or nucleoside triphosphate diphosphohydrolase. AMP is converted to adenosine by CD73, also called ecto-5'-nucleotidase [10,11], and is released by the cells of the cardiovascular system in response to various injurious stimuli or ischemic insult [12]. Although adenosine formation may be important, the formation of adenosine from ATP and AMP is limited due to the availability of CD39 and CD73 which are thus rate-limiting for extracellular adenosine formation [13]. Adenosine activates adenosine receptors on a variety of cells [10], and in the heart it plays a role in regulating, among other things, coronary blood flow, heart rate, cardiac conduction and substrate metabolism [12]. Adenosine is also known to affect several of the proposed reperfusion injury mechanisms, promoting preservation of microvascular flow, reducing reactive oxygen species (ROS) synthesis, stabilising cellular membranes, restoring calcium homeostasis and modifying the inflammatory process [14-22]. ATP, on the contrary, has been found to have cardiotoxic effects [23].

Ectonucleotidase-dependent adenosine generation has also been implicated in pre- and postconditioning related cardioprotection against ischemia-reperfusion injury [24]. The molecular mechanisms involved in preconditioning are not completely understood but are believed to involve adenosine receptor dependent activation of mitochondrial ATP-sensitive K+ (mitoK_ATP_) channels and protein kinase C (PKC) [24]. The exact relationship between mitoK_ATP_, PKC, and the end effectors remains to be clarified. However, there is evidence of cardiomyocyte modulation of sarcoplasmatic reticulum Ca^2+ ^handling, and ATP depletion with secondary effects on the mitochondrial permeability transition pore (MPTP) leading to apoptosis [12,25-29]. Similarly to preconditioning, postconditioning appears to activate signalling pathways that involve adenosine, mitoK_ATP _and PKC, with subsequent effects on MPTPs. In that respect, pre- and postconditioning seem to converge on signalling pathways that involve adenosine and are active primarily during reperfusion [30,31].

Moreover, inhibition of ATP degradation via targeted gene deletion or inhibition of CD39 and CD73 has been found to eliminate the cardioprotective effect of preconditioning [32], and increase infarct size [33]. Ectonucleotidase treatment with apyrase (CD39) has also been shown to reduce myocardial infarct size (IS) when applied prior to induction of ischemia [33].

However, apyrase treatment according to a clinically applicable protocol, with administration only after induction of ischemia, has not previously been evaluated. We therefore investigated if apyrase treatment, administered after induction of ischemia, would reduce IS and MO. Apyrase was chosen because it potentially could confer a double benefit in simultaneously generating cardioprotective adenosine and reducing cardiotoxic ATP [23].

## Methods

### Experimental preparation

16 healthy domestic male and female 40-50 kg pigs were fasted overnight with free access to water. Premedication was administered with azaperone (Stresnil Vet., Leo; Helsingborg, Sweden), 2 mg/kg intramuscularly, 30 minutes prior to the procedure. After induction of anesthesia with thiopental (Pentothal, Abbott, Stockholm, Sweden) 5-25 mg/kg, the animals were orally intubated with cuffed endotracheal tubes. Thereafter, a slow infusion of 1.25 μl/ml fentanyl (Fentanyl, Pharmalink AB, Stockholm, Sweden) in buffered glucose (25 mg/ml) was started at a rate of 1.5 ml/min and adjusted as needed. During balanced anesthesia meprobamat (Mebumal, DAK, Copenhagen, Denmark) and thiopental (Pentothal, Abbott, Stockholm, Sweden), was titrated against animal requirements with small bolus doses. Mechanical ventilation was established with a Siemens-Elema 900B ventilator in the volume-controlled mode, adjusted in order to obtain normocapnia. Initial settings were: respiratory rate of 15/min, tidal volume of 10 ml/kg and positive end-expiratory pressure of 5 cmH_2_O. The animals were ventilated with a mixture of dinitrous oxide (70%) and oxygen (30%). The pigs were continuously monitored with electrocardiogram (ECG) and intraarterial blood pressure. Heparin (200 IU/kg) was given intravenously at the start of the catheterization. An 11 F introducer sheath (Onset, Cordis Co., Miami, FL, USA) was inserted into the surgically exposed left femoral vein. A 10.7 F Celsius Control™ catheter (Innercool Therapies Inc, San Diego, CA, USA) was inserted through the sheath and positioned in the inferior vena cava with the tip of the catheter at the level of the diaphragm. Body temperature was measured with a temperature probe (TYCO Healthcare Norden AB, Solna, Sweden) placed in the distal part of the esophagus. The catheter and the temperature probe were connected to the Celsius Control console and the system was set to maintain a normal pig body temperature of 38.0°C. A 6 F introducer sheath (Onset, Cordis Co. Miami, FL, USA) was then inserted into the surgically exposed left carotid artery upon which a 6 F JL4 Wiseguide™ (Boston Scientific Scimed, Maple Grove, MN, USA) was inserted into the left main coronary artery. The catheter was used to place a 0.014-inch PT Choice™ guide wire (Boston Scientific Scimed, Maple Grove, MN, USA) into the distal portion of the left anterior descending artery (LAD). A 3.0 x 20 mm Ranger™ over-the-wire angioplasty balloon (Boston Scientific Scimed, Maple Grove, MN, USA) was then positioned in the mid portion of the LAD, immediately distal to the first diagonal branch. All radiological procedures were performed in an experimental catheterization laboratory (Shimadzu Corp., Kyoto, Japan).

In a separate group of pigs (n = 14) the general set-up of the experiment as described above, was established. However, a 0.014-inch, 12 MHz pulsed Doppler flow velocity transducer (Jometrics Flowire, Jomed NV, the Netherlands) was positioned in the mid-portion of the LAD. A 0.014-inch PT choice™ guidewire (Boston Scientific Scimed, Maple Grove, MN, USA) was thereafter inserted into the distal portion of the LAD. A 3.0 × 20 mm over-the-wire Maverick™ angioplasty balloon (Boston Scientific Scimed, Maple Grove, MN, USA) was positioned in the mid portion of the LAD, proximal to the flow velocity transducer but distal to the first diagonal branch, followed by the withdrawal of the PT choice guidewire. Continuous coronary velocity flow profiles were displayed and recorded using the Doppler flow wire connected to a FloMap monitor (Cardiometrics, Mountain View, CA, USA).

### Experimental protocol

Ischemia was induced by inflation of the angioplasty balloon for 40 min. An angiogram was performed after inflation of the balloon and before deflation of the balloon in order to verify total occlusion of the coronary vessel and correct balloon positioning. After deflation of the balloon a subsequent angiogram was performed to verify restoration of blood flow in the previously occluded artery. Twenty minutes after balloon inflation, pigs were randomized to 40 min of 1 ml/min intracoronary infusion of the ectonucleotidase apyrase (CD39, MDL number MFCD00130542, EC number 232-569-8, CAS number 9000-95-7, article number A6535, lot number 077K7016, Sigma-Aldrich, Stockholm, Sweden; 10 U/ml, n = 8) or saline (0.9 mg/ml, n = 8). According to the specification of the manufacturer, one unit of apyrase will liberate 1.0 μmole of inorganic phosphate from ATP or ADP per minute at pH 6.5 at 30°C. The infusion was made through the over-the-wire balloon, selectively into the ischemic area.

The group of animals with flow transducers was used in order to verify cardiac effects of the infusion of apyrase. The animals in the apyrase group (n = 5) received an intracoronary infusion of apyrase as decribed above during a 10 minute period of ischemia, induced by inflation of the balloon, whereafter reactive coronary hyperemia was measured and compared to controls (n = 8). Flow was measured as average peak velocity (APV) in cm/sec. In a closed chest pig model it is not possible to measure vessel diameter and doppler flow simultaneously. However, the diameter of the LAD was measured in separate pigs from both the apyrase- and control group during baseline and during reperfusion at the same angle, and was found not to increase or decrease more than 10% even during maximum reactive hyperemia. Compensation for this only resulted in very minor changes of the results and was therefore not performed.

### Imaging

*Ex vivo *imaging of the heart was undertaken according to a previous described protocol [34,35]. The MR and SPECT images were analyzed using freely available software (Segment 1.700, Medviso, Lund, Sweden, http://segment.heiberg.se) [36].

### Infarct size and microvascular obstruction assessed by *ex vivo *MRI

A gadolinium-based MRI contrast agent (Dotarem, gadoteric acid, Gothia Medical, Billdal, Sweden) was administered intravenously (0.4 mmol/kg) 30 minutes prior to explantation of the heart. The heart was removed 4 h after initiation of reperfusion. After removal, the heart was immediately rinsed in cold saline and the ventricles were filled with balloons containing deuterated water. MRI was performed using a 1.5 T Philips Intera CV MR scanner (Philips, Best, the Netherlands). T1-weighted images (time resolution = 20 ms, echo time = 3.2 ms, flip angle = 70° and 2 averages) with an isometric resolution of 0.5 mm covering the entire heart were then acquired using a quadrature head coil. The endocardial and epicardial borders of the left ventricular myocardium were manually delineated in short-axis *ex vivo *images. The volume of the left ventricular myocardium was calculated as the product of the slice thickness (cm) and the area formed by the delineated borders of the epi- and endocardium. The infarct size was determined as the volume of infarcted myocardium (cm^3^). The infarct volume was calculated as the product of the slice thickness (cm) and the area of hyperenhanced pixels (cm^2^) with a signal intensity above the infarction threshold defined as > 8 SD above the mean intensity of non-affected remote myocardium [37]. Microvascular obstruction was defined as hypointense regions in the core of the infarction which had signal intensity less than the threshold for infarction. These regions were manually included in the infarct volume. The volume of microvascular obstruction (cm^3^) was calculated as the difference between the infarct volume before and after manual inclusion of regions of microvascular obstruction. The size of microvascular obstruction was expressed as percent of the total infarct volume. The infarct size was expressed as percent of left ventricular myocardium as well as percent of the area at risk (IS/AAR) in order to adjust for any difference in AAR between the groups.

### *Ex vivo *assessment of area at risk by SPECT

Single photon emission computed tomography (SPECT) was used to assess the AAR as percent of left ventricular myocardium. Five hundred MBq of ^99 m^Tc-tetrofosmin was administered intravenously ten minutes before deflation of the angioplasty balloon. *Ex vivo *imaging was performed with a dual head camera (Skylight, Philips, Best, the Netherlands) at 64 projections (60 s per projection) with a 64 X 64 matrix and a zoom factor of 2.19, yielding a digital resolution of 4.24 X 4.24 X 4.24 mm. Iterative reconstruction using maximum likelihood-expectation maximization (MLEM) was performed with a low-resolution Butterworth filter with a cut-off frequency set to 0.6 of Nyquist and order 5.0. No attenuation or scatter correction was applied. Finally short and long-axis images were reconstructed. The endocardial and epicardial borders of the left ventricle that were manually delineated in the MR images were copied to the co-registered SPECT images. A SPECT defect was defined as a region within the MRI-determined myocardium with counts lower than the 55% of the maximum counts in the myocardium (Ugander M, Soneson H, Heiberg E et al. *A novel method for quantifying myocardial perfusion SPECT defect size by co-registration and fusion with MRI - an experimental ex vivo imaging pig heart study*. Abstract. Proceedings of the Swedish Heart Association Spring Meeting 2008).

### Calculation and statistics

Calculations and statistics were performed using the GraphPad Prism 4.0 software (GraphPad Software, Inc., La Jolla, CA, USA). Values are presented as mean ± SEM. Statistical significance was accepted when *P *< 0.05 (Mann-Whitney's test).

### Ethics

The study conforms to the Guide for the Care and Use of Laboratory Animals, US National Institute of Health (NIH Publication No. 85-23, revised 1996) and was approved by the Ethics Committee of Lund University, Sweden.

## Results

**Figure 1:**
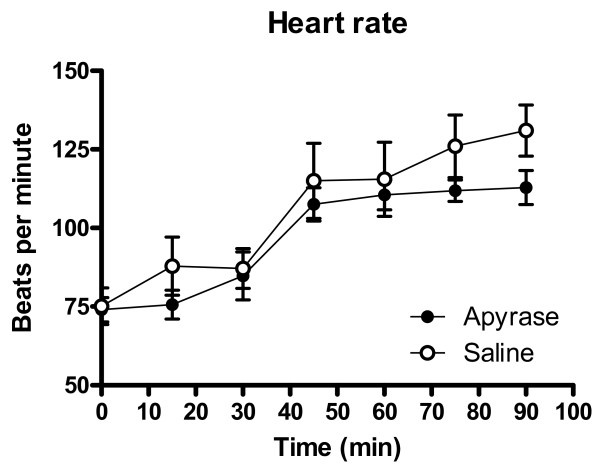
**Heart rate**. Heart rate did not differ significantly between the groups. At 90 minutes there was a trend towards a lower heart rate in the apyrase group (112 ± 5 vs 131 ± 8, p = 0.09). Error bars denote SEM.

**Figure 2:**
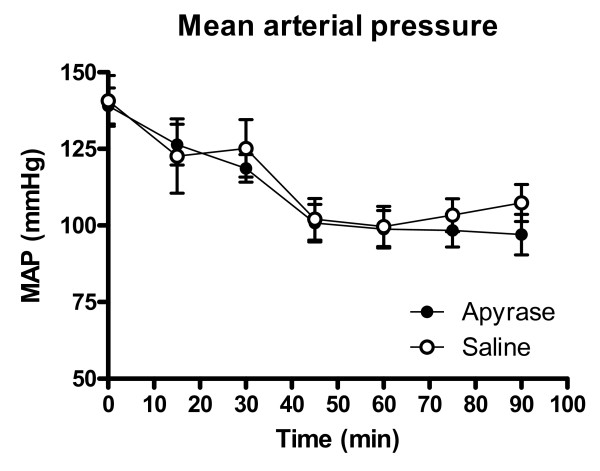
**Mean arterial pressure**. Mean arterial pressure did not differ significantly between the groups. Error bars denote SEM.

**Figure 3:**
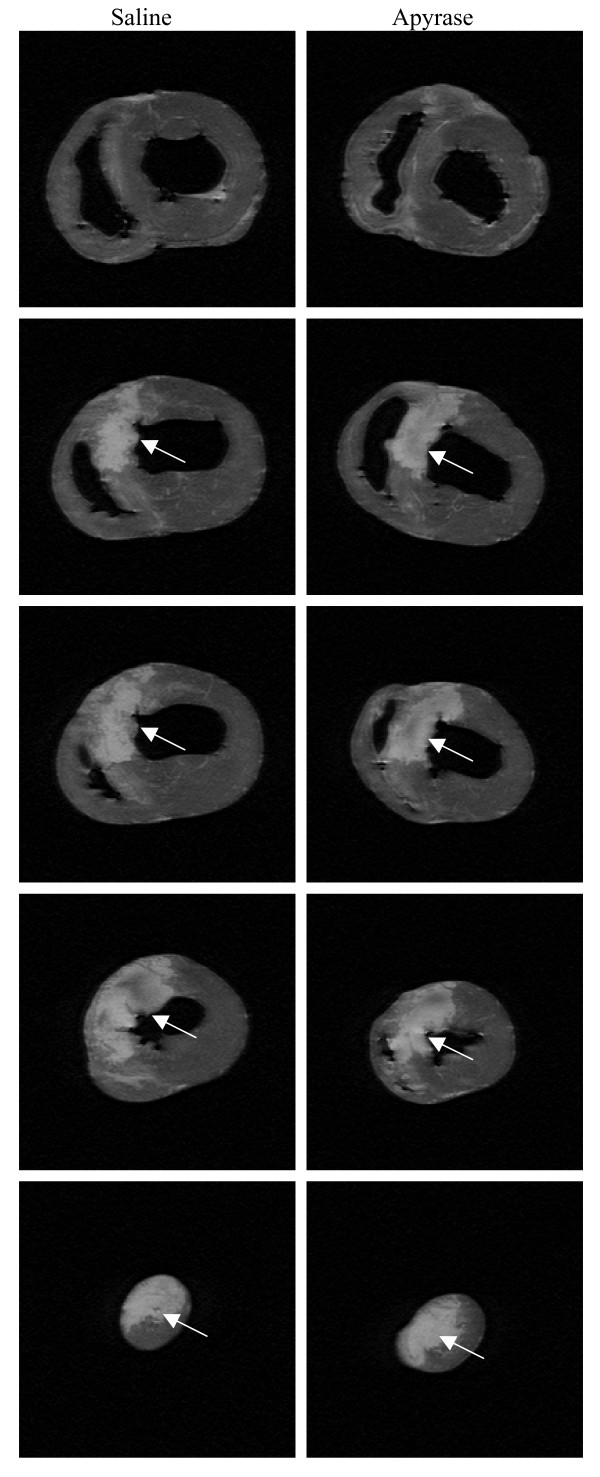
**MR images**. Delayed contrast enhanced *ex vivo *T1-weighted MR images from one animal from the saline group and one animal from the apyrase group. White areas (arrow) are infarcted. Infarct size was similar between the groups.

**Figure 4:**
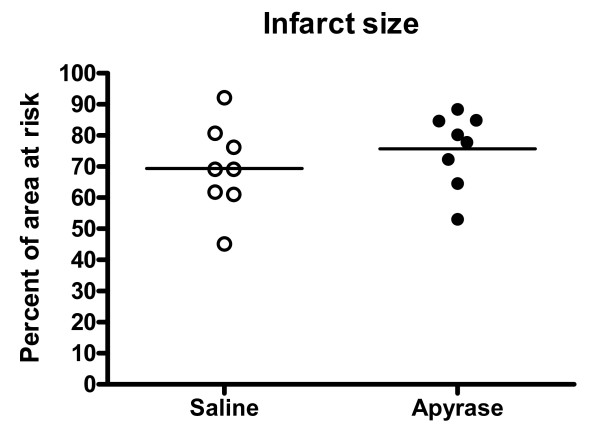
**Infarct size**. Infarct size as a percentage of area at risk from the animals in the saline group and apyrase group, respectively. No differences were seen between the groups with respect to IS/AAR. Horizontal line denotes mean.

**Figure 5:**
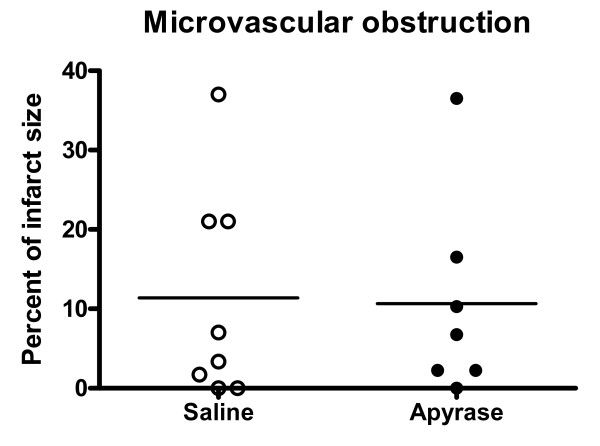
**Microvascular obstruction**. Microvascular obstruction as a percentage of infarct size from the animals in the saline group and apyrase group, respectively. No differences were seen between the groups with respect to MO. One animal is missing in the apyrase group due to technical difficulties. Horizontal line denotes mean.

**Figure 6:**
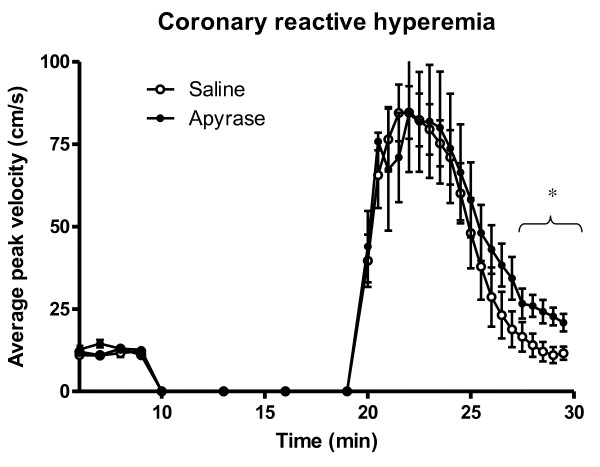
**Reactive hyperemia**. Post ischemic coronary reactive hyperemia was increased during the later phase by infusion of apyrase (p < 0.05, time-points marked by the bracket). Error bars denote SEM.

Heart rate and mean arterial pressure did not differ significantly between the groups throughout the experiment, figure 1 and 2. IS was 28.9 ± 3.1% of the left ventricular mass in the apyrase group and 27.0 ± 3.8% of the left ventricular mass in the saline group, p = NS (figure 3). AAR was 37.9 ± 3.1% of the left ventricular mass in the apyrase group and 37.8 ± 3.4% of the left ventricular mass in the saline group, p = NS. No differences were seen between the apyrase group and saline group with respect to IS/AAR (75.7 ± 4.2% vs 69.4 ± 5.0%, p = NS) or MO (10.7 ± 4.8% vs 11.4 ± 4.8%, p = NS), figure 4 and 5. pH and base excess (BE) did not differ between the groups, table 1. In the group where coronary blood flow was measured, a pronounced postischemic reactive hyperemia was observed. Infusion of apyrase caused a statistically significant increase in the later part of the hyperemic flow, figure 6.

**Table 1:** Arterial blood-gas data

	Saline	Apyrase	P-value
pH - baseline	7.50 (± 0.02)	7.49 (± 0.03)	NS
pH - 15 min	7.45 (± 0.03)	7.44 (± 0.07)	NS
pH - 45 min	7.44 (± 0.02)	7.42 (± 0.03)	NS
pH - 75 min	7.46 (± 0.02)	7.43 (± 0.03)	NS
Base excess - baseline	8.10 (± 0.52)	8.15 (± 0.78)	NS
Base excess - 15 min	5.62 (± 1.14)	6.80 (± 1.90)	NS
Base excess - 45 min	5.18 (± 1.00)	5.18 (± 1.86)	NS
Base excess - 75 min	5.05 (± 0.79)	5.05 (± 1.7)	NS

Values are expressed as mean and SEM. pH and base excess were similar between the groups throughout the experiment. The unit for base excess is mmol/L and for pH -log_10_{H^+^}

## Discussion

This study evaluates the cardioprotective effect of apyrase treatment, according to a clinically applicable protocol with administration during and after ischemia. The main findings were that IS/AAR and MO were unaffected by apyrase treatment. However, reactive hyperemia was prolonged by apyrase.

The porcine model was chosen because it more closely resembles the human pathophysiology than the rodent models and also allows for a closed chest model utilising human coronary interventional devices. A percutaneous catheter-based approach allows for induction of ischemia with minimum trauma, operation-induced stress and secondary changes in circulatory physiology. The porcine model also offers the possibility of SPECT and MRI for ischemia and infarct size evaluation. *Ex vivo *MRI allows for acquisition of high resolution images and objective semiautomatic quantification of myocardial infarction, and correlates closely to histology [35,38]. MRI and SPECT are also the gold standard methods of evaluation of ischemia and infarct size in clinical practice. In order to avoid a spontaneous variation in temperature during the experiment, a normal core body temperature of pigs (38°C) was established before induction of ischemia. A temperature difference between the groups could thus not camouflage a true effect of apyrase treatment. Temperature is known to be a major determinant of infarct size [39]. The placement of the balloon after the first diagonal branch resulted in reproducible ischemia of, on average, 38% of the left ventricle, with no difference in AAR between the groups.

There was a trend towards lower heart rate in the apyrase group that may be related to the inhibitory effect of generated adenosine on heart rate [40]. However, there were no statistically significant differences in hemodynamic parameters between the groups.

This trial was specifically aimed at evaluating the clinical applicability of apyrase treatment in the setting of ST-segment elevation myocardial infarction (STEMI). The prehospital patient delay time is often significantly longer than the in-hospital door-to-reperfusion time and the window of opportunity for cardioprotective treatment is thus limited to the later part of ischemia [41]. Consequently, apyrase was administered only during the final 20 minutes of ischemia and continued after reperfusion, in order to mimic the clinical situation. The total ischemia time of 40 min is shorter than in the typical patient with myocardial infarction, which typically experience 2-4 hours of ischemia before start of treatment. However, pigs complete myocardial infarction more rapidly than humans, and longer duration of ischemia have resulted in a large established infarct before initiation of apyrase treatment [42].

Previously, apyrase treatment has been shown to reduce IS/AAR with 43% when administered 30 minutes prior to a period of 60 minutes of ischemia in a rodent model [33]. In that study, 80 U/kg of apyrase was administered in wild-type mice. With an average mouse weight of 20 g and a percentage distribution of cardiac output with 18% going to the heart during rest [43], 0.28 U of apyrase would have been delivered to the heart. In our study, an approximately 1400 times higher dose of 400 U was infused directly into the ischemic area in order to maximize the possible cardioprotective effect of apyrase treatment at this later timepoint. The finding of prolonged coronary reactive hyperemia induced by apyrase-infusion, suggest that adenosine is generated by the apyrase-infusion. The mechanism behind reactive hyperemia is thought to be multifactorial, but adenosine has been shown to mediate the later phase of hyperemic flow via A2A receptors on smooth muscle cells [44,45]. Thus, an increased level of adenosine would be expected to increase the later phase of hyperemia, which it did in our experiment. This observation is also consistent with findings of an accelerated recovery of microvascular circulation after intracoronary infusion of adenosine as an adjunct to primary PCI [46].

The lack of cardioprotective effect of the apyrase-infusion could be explained by species-related differences in the pharmacology of adenosine receptors. It is also possible that apyrase was not able to generate enough adenosine to activate signalling via the A2B receptor, which otherwise has been able to yield cardioprotection when stimulated prior to reperfusion by the receptor agonist AMP 579 in a porcine model [47]. Indeed, the A2B receptor is known to be of lower affinity for adenosine than the A2A receptor [48], which seemed to respond with the increased flow seen at the end of reactive hyperemia in the apyrase group [44,45]. However, there is also evidence of A2A mediated cardioprotection against ischemia-reperfusion injury [12]. Theoretically, the high dose of apyrase that was used could also have a cardiotoxic effect. However, we are not aware of any such evidence in the literature. The lack of effect could also be explained by a species-related imbalance between apyrase and downstream adenosine generation by CD73 as well as by a low release of native ATP and AMP. A relative deficiency of AMP degradation capacity would be in agreement with the uncertain effect of postconditioning in pigs. Two studies of postconditioning in pig have been carried out where one was negative and the other was negative in one arm with 4 cycles of ischemia/reperfusion and positive in another arm with 8 cycles [49,50]. In another study in a porcine model, preconditioning was found to be cardioprotective [51]. In that study, cardiac adenosine levels were measured in the preconditioning group and found to be increased during preconditioning but attenuated during ischemia. In the control group, the adenosine concentration was elevated during ischemia, as opposed to the finding in the preconditioning group [51]. The increased adenosine level during ischemia seen in the control group is in agreement with the experimental protocol in our study. This clearly supports the conclusion that the apyrase infusion must be initiated prior to the induction of ischemia in order to generate a cardioprotective effect. Thus, it is possible that apyrase treatment could prove to be cardioprotective if initiated earlier than in the current study, but it would be of little interest in clinical practice. These findings may also imply that other triggers of pre- and postconditioning, such as bradykinin, opioids, nitric oxide and reactive oxygen species [30,31,52], could be of greater importance than adenosine in the setting of pre- and postconditioning. Furthermore, these factors may explain why apyrase infusion has been found to reduce infarct size in a rodent model but not in our porcine model.

It may be of interest to study whether or not apyrase treatment is cardioprotective in the setting of non-ST elevation acute coronary syndrome, where ischemia may be less sudden in onset and time to coronary intervention longer. In this less sudden setting the stimulatory effect of adenosine on angiogenesis may prove to be effective in tissue protection, in addition to the above mentioned mechanisms of effect [53]. Further research is warranted and the CD39 knockout pig under development may be used to further advance the knowledge in the field.

## Limitations

Histopathological evaluation was not performed. MRI correlates excellent with histological evaluation and this would not affect the outcome parameters. However, histology could have added information about changes at the cellular and molecular levels which could be early indicators of possible beneficial effects of apyrase. Moreover, levels of adenosine and apyrase were not measured, in part because the protocol was maximized regarding intracoronary apyrase administration. A biologically relevant effect of the apyrase infusion was instead confirmed by the finding of an increased reactive hyperemia. However, information about the amount of adenosine that was generated could have conferred additional valuable information.

## Conclusion

In summary, apyrase treatment according to a clinically applicable protocol, with administration of apyrase only after induction of ischemia, does not reduce myocardial infarct size or microvascular obstruction in the setting of STEMI.

## Competing interests

The authors declare that they have no competing interests.

## Authors' contributions

The authors have contributed as follows: Conception and design (DE and JVDP), animal experimentation (JVDP, SK, MIG, GO and MG), image design and acquisition (MU, MK, AO and HA), analysis and interpretation (JVDP and DE), drafting of the manuscript (JVDP and DE), critical revision for important intellectual content (all authors), final approval of the manuscript (all authors).

## Pre-publication history

The pre-publication history for this paper can be accessed here:

http://www.biomedcentral.com/1471-2261/10/1/prepub
